# Supplementary material for: Cognitive flexibility and brain network energy in healthy aging: An allostatic perspective from the SENECA model
Source: Imaging Neurosci (Camb). 2026 Jan 12;4:IMAG.a.1091. doi: 10.1162/IMAG.a.1091 (PMC12797148; doi:10.1162/IMAG.a.1091)
Supplement: Supplementary Material [file IMAG.a.1091_supp.pdf]

Supplementary materials

Description of the 8 neuropsychological tests used to assess lexical production. Please refer to original CamCAN articles for more information on behavioral datasets (Cam-CAN et al., 2014; Taylor et al., 2017).

Cattell: *Cattell Culture Fair Test: Complete nonverbal puzzles involving series completion, classification, matrices, and conditions* (Cattell & Cattell, 1960).

Hotel Task: *Perform simulated tasks of a hotel manager: write customer bills, sort money, proofread adverts, sort playing cards, alphabetize a list of names. Total time must be allocated equally between tasks; there is not enough time to complete any task* (Shallice & Burgess, 1991). ***In this study, we applied a log transformation and subtracted it from 1 to stay consistent with a decrease as age increases (i.e., 1-log(x)).***

Picture Naming: *Name the pictured object presented alone (baseline), then when preceded by a prime object that is phonologically related (one or two initial phonemes), semantically related (low or high relatedness), or unrelated* (Clarke et al., 2013).

Proverb: *Read and interpret three English proverbs* (Huppert et al., 1994).

Sentence Comprehension: *Listen to and judge the grammatical acceptability of partial auditory sentences that begin with an ambiguous sentence stem (e.g., "Tom noticed that landing planes…") followed by a disambiguating continuation word (e.g., "are") in a different voice. Ambiguity is either semantic or syntactic, with empirically determined dominant and subordinate interpretations* (Rodd et al., 2010).

Story Recall: *Listen to a short story, recall freely immediately after, then again after a delay, and finally answer recognition memory questions* (Tulsky et al., 2003). *Delayed recall measure used here.*

Tip-of-the-Tongue (ToT): *Participants are asked to name famous faces and indicate if they know/don't know/or have a ToT* (Brown & McNeill, 1966). ***In this study, we subtracted the score from 1 to stay consistent with a decrease as age increases (i.e., 1-x).***

Verbal Fluency: *Mean of letter (phonemic) fluency and animal (semantic) fluency task. For the phonemic fluency task, participants have 1 minute to generate as many words as possible beginning with the letter 'p'. For the semantic fluency task, participants have 1 minute to generate as many words as possible in the category "animals"* (Lezak et al., 2012).

Supplementary results

Three network configurations	Age of inflection in the trajectory (2nd derivative)	F (age)	edf	signed partial R2	pFDR (q < 0.05)
	**Age-related changes in between-network flexibility**				
('Auditory', 'CON', 'SMN')	52.36	31.50	1.95	0.09	0
('DMN', 'FPN', 'Visual_1')	NA	22.03	1.55	-0.06	0
('CON', 'FPN', 'Visual_1')	NA	33.88	1.00	-0.05	0
('Auditory', 'Language', 'SMN')	52.36	17.50	1.90	0.05	0
('Auditory', 'DMN', 'FPN')	NA	29.09	1.02	-0.05	0
('Auditory', 'SMN', 'Visual_2')	52.36	13.96	1.93	0.04	0
('DAN', 'DMN', 'FPN')	52.36	14.72	1.84	-0.04	0
('Auditory', 'SMN', 'Visual_1')	52.36	13.64	1.94	0.04	0
('CON', 'FPN', 'Visual_2')	NA	26.26	1.00	-0.04	0
('Auditory', 'DAN', 'SMN')	52.36	12.71	1.94	0.04	0

Pair of networks	Age of inflection in the trajectory (2nd derivative)	F (age)	edf	signed partial R2	pFDR (q < 0.05)
	**Age-related changes in between-network flexibility**				
('Auditory', 'SMN')	52.36	53.17	1.94	0.14	0
('Auditory', 'CON')	52.36	30.00	1.96	0.09	0
('SMN', 'Visual_2')	52.36	16.86	1.93	0.05	0
('SMN', 'Visual_1')	52.36	15.92	1.95	0.05	0
('CON', 'SMN')	52.36	12.88	1.91	0.04	0
('Visual_1', 'Visual_2')	52.36	12.73	1.91	0.04	0
('Auditory', 'Language')	52.36	12.81	1.90	0.04	0
('Language', 'SMN')	NA	13.60	1.75	0.04	0
('DMN', 'FPN')	52.36	12.35	1.81	-0.04	0
('FPN', 'Visual_1')	NA	16.42	1.19	-0.03	0

Network	Age of inflection in the trajectory (2nd derivative)	F (age)	edf	signed partial R2	pFDR (q < 0.05)
	**Age-related changes in within-network energy**				
SMN	52.36	38.97	1.92	0.11	0
Auditory	52.36	37.91	1.81	0.10	0
Language	NA	40.35	1.46	0.09	0
Visual_2	52.36	31.69	1.85	0.09	0
CON	52.36	19.80	1.94	0.06	0
Visual_1	52.36	12.84	1.93	0.04	0
DAN	NA	8.75	1.66	0.02	0

Table S1. GAM statistics at the network level. We report the significant trajectories with the 10 largest positive age-related effect

Salient energy changes - LC1				
**BSR**	**Region**	**Substrate**	**Location**	**Network**
**11.03**	L_V4	Fourth_Visual_Area_L	Early_Visual	Visual_2
**9.99**	L_V3	Third_Visual_Area_L	Early_Visual	Visual_2
**9.11**	L_OP4	Area_OP4-PV_L	Posterior_Opercular	SMN
**8.86**	R_V3	Third_Visual_Area_R	Early_Visual	Visual_2
**8.69**	R_3b	Primary_Sensory_Cortex_R	Somatosensory_and_Motor	SMN
**8.55**	L_OP1	Area_OP1-SII_L	Posterior_Opercular	SMN
**8.34**	L_PFcm	Area_PFcm_L	Early_Auditory	CON
**8.32**	L_3b	Primary_Sensory_Cortex_L	Somatosensory_and_Motor	SMN
**8.30**	R_V4	Fourth_Visual_Area_R	Early_Visual	Visual_2
**8.29**	R_OP2.3	Area_OP2-3-VS_R	Posterior_Opercular	SMN
**-12.64**	R_23d	Area_23d_R	Posterior_Cingulate	DMN
**-11.93**	R_8Ad	Area_8Ad_R	Dorsolateral_Prefrontal	DMN
**-10.92**	L_Hipp	Hippocampus_L	Medial_Temporal	DMN
**-10.87**	L_8Ad	Area_8Ad_L	Dorsolateral_Prefrontal	DMN
**-10.78**	L_23d	Area_23d_L	Posterior_Cingulate	DMN
**-10.64**	R_Hipp	Hippocampus_R	Medial_Temporal	DMN
**-10.63**	L_31a	Area_31a_L	Posterior_Cingulate	DMN
**-10.27**	L_POS2	Parieto-Occipital_Sulcus_Area_2_L	Posterior_Cingulate	FPN
**-9.94**	R_s6.8	Superior_6-8_Transitional_Area_R	Dorsolateral_Prefrontal	FPN
**-9.76**	R_POS2	Parieto-Occipital_Sulcus_Area_2_R	Posterior_Cingulate	FPN

Salient energy changes - LC2				
**BSR**	**Region**	**Substrate**	**Location**	**Network**
**-6.19**	R_A5	Auditory_5_Complex_R	Auditory_Association	Language
**-5.62**	L_A4	Auditory_4_Complex_L	Auditory_Association	Auditory
**-5.07**	R_23c	Area_23c_R	Paracentral_Lobular_and_Mid_Cingulate	CON
**-4.92**	R_4	Primary_Motor_Cortex_R	Somatosensory_and_Motor	SMN
**-4.79**	L_6a	Area_6_anterior_L	Premotor	DAN
**-4.71**	L_23c	Area_23c_L	Paracentral_Lobular_and_Mid_Cingulate	CON
**-4.68**	L_d32	Area_dorsal_32_L	Anterior_Cingulate_and_Medial_Prefrontal	DMN
**-4.62**	L_OP4	Area_OP4-PV_L	Posterior_Opercular	SMN
**-4.62**	R_p24pr	Area_Posterior_24_prime_R	Anterior_Cingulate_and_Medial_Prefrontal	CON
**-4.62**	R_V3B	Area_V3B_R	Dorsal_Stream_Visual	Visual_2

Table S2. Salient features for each latent component of the PLS model. We report the salient trajectories with the 10 largest negative and the 10 largest positive age-related effect

Robustness analysis

Method 1: Null models preserving spatial and temporal autocorrelation (Shinn et al., 2023) retained 70% (SD: 14%) of pairwise functional connections on average across all subjects. Significantly more connections were preserved in older adults, particularly beyond midlife ($F = 67.29$, $p < .001$, $edf = 1.89$, signed partial $R^2 = -.17$). This is indicative of denser, noise-resistant network connectivity patterns in older adulthood.

No lifespan change in overall flexibility emerges after density correction (ROPE fully covers the 89% HDI of posterior samples), suggesting that denser functional connectomes in aging do not compromise global energy levels. Substantial changes in between vs. within network flexibility ($F = 24.92$, $p < .001$, $edf = 1.88$, signed partial $R^2 = -.07$), with a similar inflection point at age 52, confirm our initial report in the main text. Global positive efficiency rises linearly but negligibly ($p = 0.03$, $edf = 1$, partial $R^2 < .01$), while negative efficiency shows halved effect size compared to our initial report ($F = 2.89$, $p = .04$, $edf = 1.82$, partial $R^2 = .01$) with significance limited to early-to-midlife drop (see Figure S1). This suggests that the extra preserved edges likely bolster cooperation (positive connectivity) and, conversely, that the reported maturation of antagonistic network organization in older adulthood could largely be attributed to artifacts driven by spatial and temporal signal dependencies.

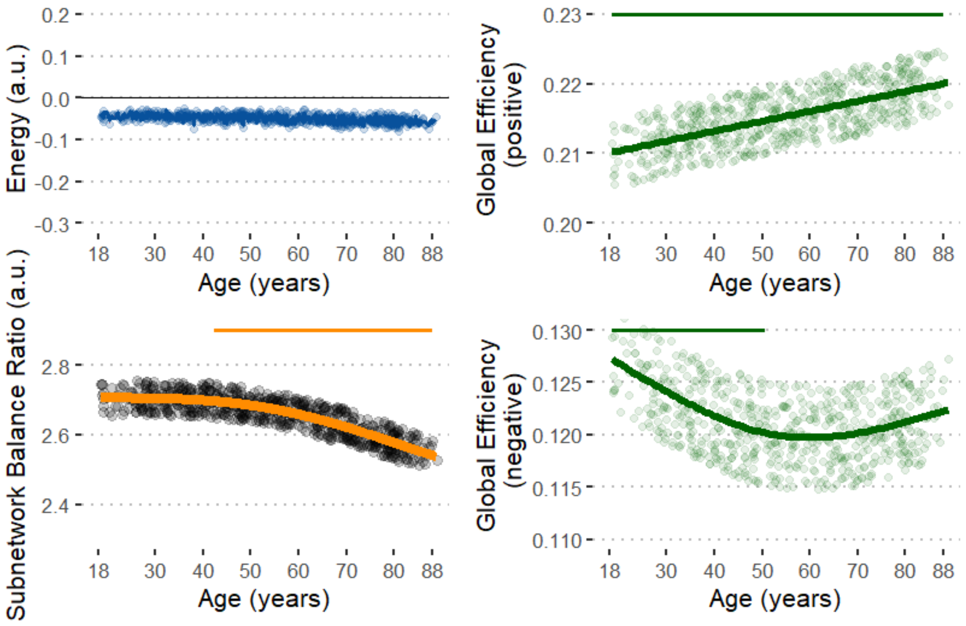

Figure S1. Thresholding method 1 – Partial replication of the results illustrated in Figure 2

We replicate the results reported in section 3.2, observing similar changes in between-network and within-network flexibility across the lifespan (see Table S3).

Age-related changes in between-network flexibility					
Three network configurations	Age of inflection in the trajectory (2nd derivative)	F (age)	edf	signed partial R2	pFDR (q < 0.05)
('Auditory', 'CON', 'SMN')	52.36	18.23	1.95	0.06	0
('Auditory', 'DMN', 'FPN')	NA	19.42	1.66	-0.05	0
('DMN', 'FPN', 'Visual_1')	52.36	16.55	1.81	-0.05	0
('CON', 'FPN', 'Visual_1')	NA	29.57	1.00	-0.05	0
('DAN', 'DMN', 'FPN')	52.36	13.95	1.92	-0.04	0
('Auditory', 'FPN', 'Visual_2')	NA	22.50	1.00	-0.03	0
('CON', 'FPN', 'Visual_2')	NA	15.25	1.28	-0.03	0
('Auditory', 'FPN', 'Visual_1')	NA	19.21	1.00	-0.03	0
('CON', 'DMN', 'FPN')	52.36	10.03	1.83	-0.03	0
('Auditory', 'DAN', 'SMN')	52.36	8.85	1.94	0.03	0

Age-related changes in between-network flexibility					
Pair of networks	Age of inflection in the trajectory (2nd derivative)	F (age)	edf	signed partial R2	pFDR (q < 0.05)
('Auditory', 'SMN')	52.36	25.64	1.93	0.08	0.00
('Auditory', 'CON')	52.36	16.84	1.96	0.05	0.00
('DMN', 'FPN')	52.36	12.94	1.91	-0.04	0.00
('FPN', 'Visual_1')	NA	12.01	1.64	-0.03	0.00
('Auditory', 'DMN')	NA	13.92	1.18	-0.03	0.00
('SMN', 'Visual_1')	52.36	8.21	1.94	0.03	0.00
('SMN', 'Visual_2')	52.36	6.92	1.92	0.02	0.01
('Auditory', 'FPN')	NA	14.08	1.00	-0.02	0.00
('CON', 'SMN')	52.36	6.69	1.91	0.02	0.01
('DAN', 'DMN')	52.36	6.47	1.90	-0.02	0.01

Age-related changes in within-network flexibility					
Network	Age of inflection in the trajectory (2nd derivative)	F (age)	edf	signed partial R2	pFDR (q < 0.05)
SMN	52.36	13.50	1.90	0.04	0.00
Auditory	NA	15.24	1.55	0.04	0.00
Language	NA	21.14	1.00	0.03	0.00
CON	52.36	9.93	1.93	0.03	0.00
Visual_2	NA	10.40	1.74	0.03	0.00
Visual_1	52.36	4.34	1.90	0.02	0.02
DMN	NA	5.48	1.61	-0.01	0.02

Table S3. Thresholding method 1 - GAM statistics at the subnetwork level

We also replicate the results reported in section 3.3, highlighting the PCC as a crucial DMN-FPN interface for flexibility, and its heterogeneous role within the DMN across the lifespan.

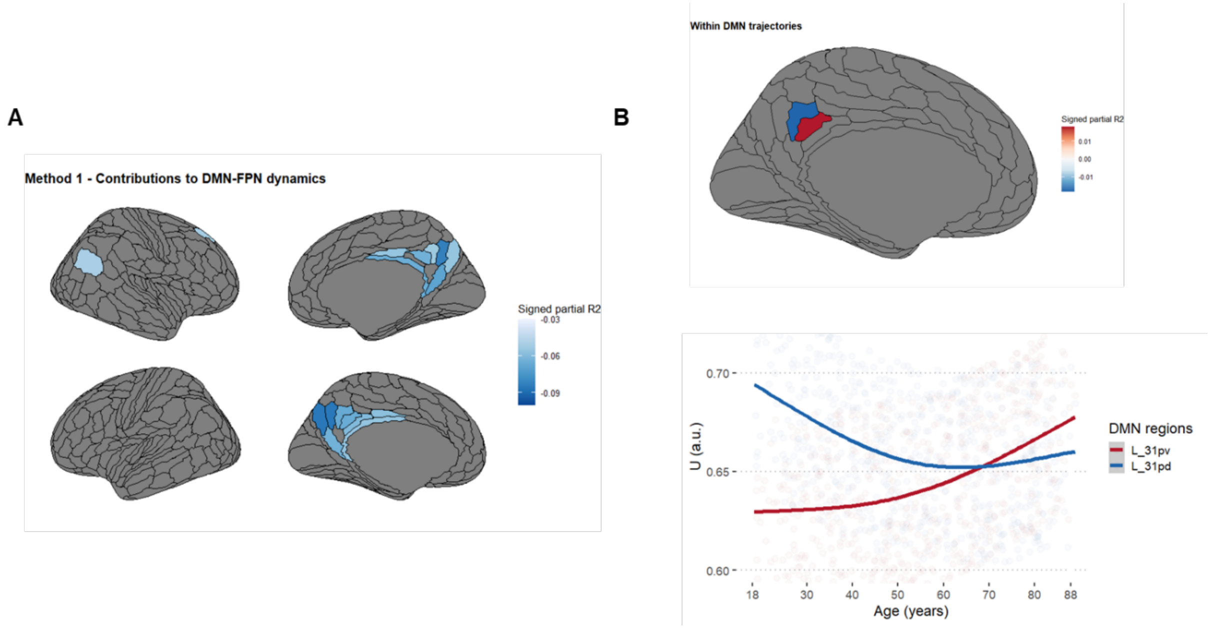

Figure S2. Thresholding method 1 - Regions contributing to changes in (A) DMN-FPN and (B) within DMN flexibility

Method 2: The correlation screening approach (Lbath et al., 2024) retained 18.4% (SD: 6%) of pairwise functional connections on average across all subjects, far sparser than Method 1's 70%, with no evidence for age-related density differences (p = .55).

No lifespan change in overall flexibility emerges after density correction (ROPE fully covers the 89% HDI of posterior samples), suggesting that denser functional connectomes in aging do not compromise global energy levels. Substantial changes in between vs. within network flexibility (F = 74.99, p < .001, edf = 1.94, signed partial R^2 = - .19), with a similar inflection point at age 52, confirm our initial report in the main text. Global positive efficiency declines linearly (p < .01 ; edf = 1, partial R^2 < .012), while negative efficiency shows a smaller effect size compared to our initial report (F = 4, p = .02, edf = 1.87, partial R^2 = .014) and retains a similar non-linear trajectory (see Figure S2).

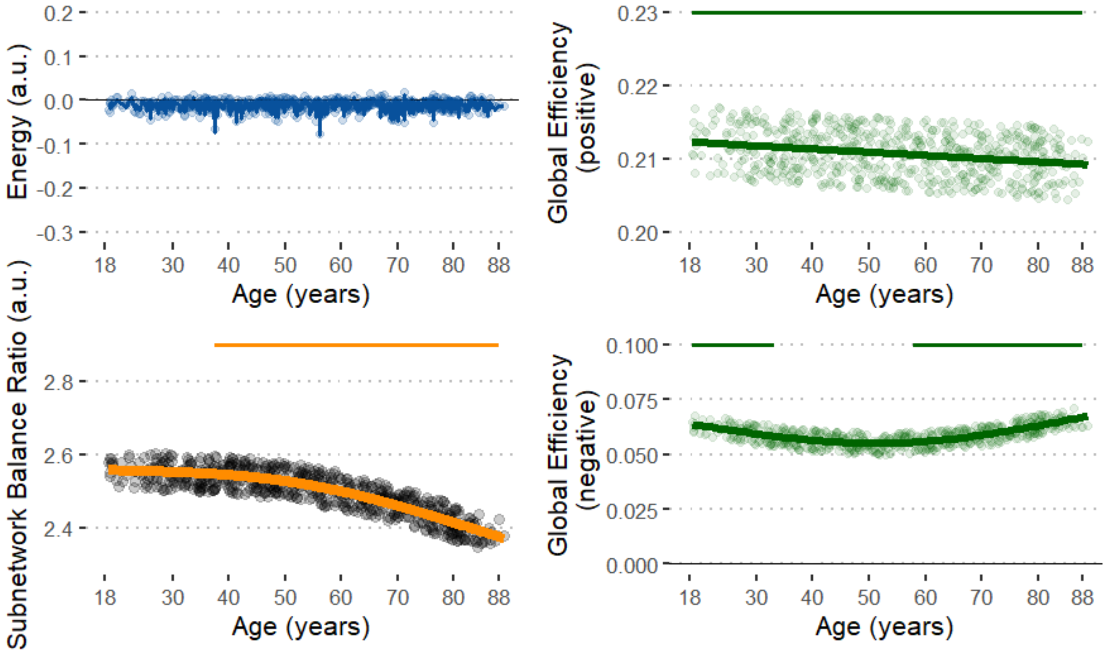

Figure S3. Thresholding method 2 – Partial replication of the results illustrated in Figure 2

We replicate the results reported in section 3.2, observing similar changes in between-network and within-network flexibility across the lifespan (see Table S4).

	Age-related changes in between-network flexibility				
Three network configurations	Age of inflection in the trajectory (2nd derivative)	F (age)	edf	signed partial R2	pFDR (q < 0.05)
('Auditory', 'CON', 'SMN')	52.36	31.90	1.95	0.09	0
('SMN', 'Visual_1', 'Visual_2')	52.36	20.06	1.96	0.06	0
('DAN', 'DMN', 'FPN')	52.36	19.91	1.94	-0.06	0
('Auditory', 'SMN', 'Visual_1')	52.36	14.35	1.95	0.04	0
('CON', 'DMN', 'FPN')	52.36	14.72	1.89	-0.04	0
('Auditory', 'Language', 'SMN')	52.36	14.28	1.91	0.04	0
('Auditory', 'DMN', 'FPN')	NA	15.23	1.60	-0.04	0
('Auditory', 'SMN', 'Visual_2')	52.36	11.08	1.93	0.03	0
('DMN', 'FPN', 'Visual_1')	NA	15.39	1.30	-0.03	0
('DAN', 'SMN', 'Visual_2')	52.36	9.45	1.93	0.03	0

	Age-related changes in between-network flexibity				
Pair of networks	Age of inflection in the trajectory (2nd derivative)	F (age)	edf	signed partial R2	pFDR (q < 0.05)
('Auditory', 'SMN')	52.36	45.36	1.94	0.13	0
('SMN', 'Visual_1')	52.36	24.04	1.96	0.07	0
('Auditory', 'CON')	52.36	22.99	1.96	0.07	0
('SMN', 'Visual_2')	52.36	20.64	1.95	0.06	0
('CON', 'SMN')	52.36	20.39	1.90	0.06	0
('Visual_1', 'Visual_2')	52.36	18.36	1.93	0.05	0
('DMN', 'FPN')	52.36	12.83	1.93	-0.04	0
('Language', 'SMN')	NA	13.98	1.76	0.04	0
('Auditory', 'DMN')	NA	12.98	1.65	-0.03	0
('DAN', 'Visual_2')	52.36	11.60	1.80	0.03	0

	Age-related changes in within-network flexibility				
Network	Age of inflection in the trajectory (2nd derivative)	F (age)	edf	signed partial R2	pFDR (q < 0.05)
SMN	52.36	52.34	1.92	0.14	0.00
Language	NA	77.38	1.00	0.11	0.00
Visual_2	52.36	40.15	1.86	0.11	0.00
Auditory	NA	37.42	1.73	0.10	0.00
CON	52.36	18.83	1.89	0.05	0.00
Visual_1	52.36	15.14	1.92	0.05	0.00
FPN	52.36	9.22	1.86	0.03	0.00
DAN	NA	11.24	1.01	0.02	0.00
DMN	52.36	3.69	1.83	-0.01	0.04

Table S4. Thresholding method 2 - GAM statistics at the subnetwork level

As mentioned in the main text (section 3.4), region level analyses could not be reliably conducted after thresholding matrices using the correlation screening approach (Method 2).

Synthesis

Both Method 1 (spatial/temporal autocorrelation nulls) and Method 2 (correlation screening) validate our core findings: global flexibility remains stable across lifespan after density correction, while midlife (age 52) marks a shift from inter-network to intra-network flexibility. Energy measures from structural balance theory prove resilient to thresholding variations, unlike graph-theoretic efficiency metrics: Older adults' anti-correlated activity appears sensitive to autocorrelation-driven noise (Method 1) but is resilient to intra-regional aggregation biases (Method 2). Conversely, Method 2 appears to be more stringent on correlated activity, while Method 1 is more permissive.

Overall, these supplementary results underscore (i) the invariance of energy-based metrics to different thresholding schemes, and (ii) the complementarity of thresholding methods for examining global efficiency: Method 1 and Method 2 better correct for spurious anti-correlations (negative) and correlations (positive), respectively.

References

Brown, R., & McNeill, D. (1966). The "tip of the tongue" phenomenon. *Journal of Verbal Learning and Verbal Behavior*, *5*(4), 325-337. https://doi.org/10.1016/S0022-5371(66)80040-3

Cam-CAN, Shafto, M. A., Tyler, L. K., Dixon, M., Taylor, J. R., Rowe, J. B., Cusack, R., Calder, A. J., Marslen-Wilson, W. D., Duncan, J., Dalgleish, T., Henson, R. N., Brayne, C., & Matthews, F. E. (2014). The Cambridge Centre for Ageing and Neuroscience (Cam-CAN) study protocol : A cross-sectional, lifespan, multidisciplinary examination of healthy cognitive ageing. *BMC Neurology*, *14*(1), 204. https://doi.org/10.1186/s12883-014-0204-1

Cattell, R. B., & Cattell, A. K. (1960). Measuring intelligence with the culture fair tests. *Institute for Personality and Ability Testing*.

Clarke, A., Taylor, K. I., Devereux, B., Randall, B., & Tyler, L. K. (2013). From Perception to Conception : How Meaningful Objects Are Processed over Time. *Cerebral Cortex*, *23*(1), 187-197. https://doi.org/10.1093/cercor/bhs002

Huppert, F. A., Brayne, C., & O'Connor, D. W. (Éds.). (1994). *Dementia and normal aging*. Cambridge Univ. Press.

Lbath, H., Petersen, A., & Achard, S. (2024). Large-scale correlation screening under dependence for brain functional connectivity network inference. *Statistics and Computing*, *34*(2), 90. https://doi.org/10.1007/s11222-024-10411-x

Lezak, M. D., Howieson, D. B., Bigler, E. D., & Tranel, D. (2012). *Neuropsychological assessment* (Fifth edition). Oxford University Press.

Rodd, J. M., Longe, O. A., Randall, B., & Tyler, L. K. (2010). The functional organisation of the fronto-temporal language system : Evidence from syntactic and semantic ambiguity. *Neuropsychologia*, *48*(5), 1324-1335. https://doi.org/10.1016/j.neuropsychologia.2009.12.035

Shallice, T., & Burgess, P. W. (1991). DEFICITS IN STRATEGY APPLICATION FOLLOWING FRONTAL LOBE DAMAGE IN MAN. *Brain*, *114*(2), 727-741. https://doi.org/10.1093/brain/114.2.727

Shinn, M., Hu, A., Turner, L., Noble, S., Preller, K. H., Ji, J. L., Moujaes, F., Achard, S., Scheinost, D., Constable, R. T., Krystal, J. H., Vollenweider, F. X., Lee, D., Anticevic, A., Bullmore, E. T., & Murray, J. D. (2023). Functional brain networks reflect spatial and temporal autocorrelation. *Nature Neuroscience*, *26*(5), 867-878. https://doi.org/10.1038/s41593-023-01299-3

Taylor, J. R., Williams, N., Cusack, R., Auer, T., Shafto, M. A., Dixon, M., Tyler, L. K., Cam-CAN, & Henson, R. N. (2017). The Cambridge Centre for Ageing and Neuroscience (Cam-CAN) data repository : Structural and functional MRI, MEG, and cognitive data from a cross-sectional adult lifespan sample. *NeuroImage*, *144*, 262-269. https://doi.org/10.1016/j.neuroimage.2015.09.018

Tulsky, D. S., Chiaravalloti, N. D., Palmer, B. W., & Chelune, G. J. (2003). The Wechsler Memory Scale, Third Edition. In *Clinical Interpretation of the WAIS-III and WMS-III* (p. 93-139). Elsevier. https://doi.org/10.1016/B978-012703570-3/50007-9
